# Effectiveness and efficiency of integrated mental health care programmes in Germany: study protocol of an observational controlled trial

**DOI:** 10.1186/1471-244X-14-163

**Published:** 2014-06-04

**Authors:** Annabel Sandra Stierlin, Katrin Herder, Marina Julia Helmbrecht, Stefanie Prinz, Julia Walendzik, Marco Holzmann, Thomas Becker, Matthias Schützwohl, Reinhold Kilian

**Affiliations:** 1 Department of Psychiatry II, Ulm University, Bezirkskrankenhaus Günzburg, Germany; 2 Department of Psychiatry and Psychotherapy, University Hospital Carl Gustav Carus Dresden, TU Dresden, Germany

**Keywords:** Integrated care, Mental illness, Depression, Schizophrenia, Quality of life, Empowerment, Cost-effectiveness

## Abstract

**Background:**

Since 2009 some German health insurance companies have implemented integrated mental health care services along the principles of assertive community treatment in collaboration with local mental health service providers across Germany. Focus of this study is the analysis of effectiveness and cost-effectiveness of this integrated care programme compared to care as usual in routine care surroundings in five regions in Germany.

**Methods:**

In this 18-month multi-centre observational trial 250 patients enrolled in an integrated mental health care programme and 250 patients who receive treatment as usual from five catchment areas will be included. In addition, in each group about 125 relatives of the participating patients will be included. The primary outcome criterion is the improvement of empowerment; secondary outcomes are subjective quality of life, functional impairment and costs of illness. Data will be collected at baseline and three follow-ups after 6, 12 and 18 months. Data will be analysed by means of mixed effects regression models. Propensity score methods are used for selection bias control.

**Discussion:**

Study results are expected to provide information about how integrated care programmes in their present form contribute to the improvement of mental health care. In addition, the study will provide hints to weaknesses of the current integrated care programme and options to overcome them. The major strengths of this study are the real-world character of the study intervention with a simultaneous high level of academic rigour. However, the fact that patients are not randomised to study groups and that there is no blinding might limit the study.

**Trial registration:**

German Clinical Trial Register DRKS00005111.

## Background

Contemporary treatment of people with permanent severe mental illness is not only aimed at the control of symptoms of disease but also aims at the empowerment of patients by increasing their capacities for a largely independent lifestyle and comprehensive social and professional inclusion. For this purpose adequate mental health care requires a broad spectrum of medical and psychosocial services. In order to keep the level of psychiatric inpatient treatment and institutional care as low as possible these services should be provided by multi-professional community mental health teams organized according to the principles of Assertive Community Treatment (ACT).

The concept of ACT has already been developed and tested in the 1970s in the USA. In line with the ACT model, Stein and Santos [1] developed a concept for a comprehensive multi-professional psychiatric treatment programme in the immediate residential environment of the patient which still serves as a model for community mental health interventions across the world. Subsequently, several modifications of the ACT concept, as intensive case management (ICM), community mental health teams (CMHT), and home treatment (HT) were developed to account for specific requirements and conditions of the respective structures of psychiatric care.

In a systematic review on the effectiveness and efficiency of these treatment approaches for the period between 1979 and 2003, Roberts et al. evaluated a total of 48 publications written in English, of which 18 were from the United States and 15 from the United Kingdom. Twelve of the studies examine the cost-effectiveness of ACT in comparison to the respective psychiatric standard care during the period 1972 to 1996, the others focus on case management, community psychiatric nurses and other forms of community psychiatric care [2]. The authors conclude on the basis of the included studies – as already done in a previous review [3] - that the investigated ACT programmes, with few exceptions, prove to be more efficient compared to inpatient treatment [2]. Rosen et al. [4] and Knapp et al. [5] came to a similar conclusion. For case management and intensive case management programmes, the authors concluded that case management compared with standard inpatient treatment is cheaper and more effective, but there are no differences compared to standard outpatient treatment [2]. Roberts et al. claim that data are inconclusive for the other treatments which were investigated within their review [2].

A Cochrane review by Marshall and Lockwood [6] of ACT for people with severe mental disorders demonstrated benefits of ACT compared to standard care with respect to hospital admissions, length of hospital stay and, as a consequence, reduced costs of hospital care. Even if total costs are not lowered by ACT, patients benefit from the intervention due to better accommodation status, more employment and higher satisfaction scores. Furthermore a lower loss-to-follow up-rate is reported. Mental state and social functioning are not affected. ACT is more effective even compared to case management as regards duration of hospitalisation and costs of hospital care [6].

These results are largely in agreement with the results of another Cochrane review of intensive case management [7]. This review planned to replace the previous one written by Marshall and Lockwood. However, as the new review focuses on intensive case management (ICM), which, in this case, is defined as ACT and CM in combination with a caseload less than 20 [7], the older review remains crucial concerning ACT itself. In general, the updated review reported fewer effects than the former one. For example, there is no longer a benefit as regards employment status, and data for cost reduction are inconclusive. Burns et al. [8] assume that the inconsistent effects over recent years might be explained by differences in trial contexts. In the meantime, welfare systems have developed a number of interventions with a view to reducing hospitalisation. Thus, control groups might be contaminated with other care services and consequently, effect sizes could be reduced. Burns et al. showed, in a systemic review including 29 trials, that the more ICM is adherent to the ACT model, the lower is duration of hospitalisation. Moreover, benefits regarding use of hospital care are more pronounced in patients with a high baseline level of hospitalisation. As new welfare systems generally aim at lower reliance on hospitals, use of hospital care at baseline is reduced in newer studies and consequently, significant reduction in costs for hospital care and also in total costs is more difficult to obtain. Thus, clinical benefits become more and more important to justify the implementation of ACT services [3].

In summary, there is very good evidence for the effectiveness of ACT compared to inpatient treatment and standard care, because it achieves better results in terms of reducing social impairments at comparable costs. However, effectiveness and cost-effectiveness of each community mental health care programme has to be evaluated in its special setting both in terms of real implementation and in terms of patients’ characteristics. Attention must be paid to unchanged costs not outweighing clinical and social benefits as in the end cost-effectiveness is what really counts.

### The situation of mental health care services in Germany

Germany is one of the countries with highest expenditure for mental health care in the world. However, in contrast to other western European countries, psychiatric treatment in Germany is still mainly provided by psychiatric hospitals, outpatient clinics and office based psychiatrists and only rarely by community mental health teams. As mental health policy, except the provision of pharmaceutical treatment, is the responsibility of the federal states, no national mental health plan exists. Therefore, community mental health care systems vary widely with regard to conceptual, organisational and economic conditions across the country. Moreover, the fact that different components of community mental health care are funded by different payers (and on different legal bases) hampers coordination and integration of services.

Meanwhile an increasing number of experts suspect that the lack of national standards for community mental health care provision leads to deficits in service effectiveness and efficiency. As the only possibility to directly influence the design of community mental health care, the federal government, in recent years, has introduced several changes to the Social Security Code which forms the legal basis for health care financing. These legal changes allowed health insurance companies to enter into contracts with medical or non-medical mental health service providers for the financing of complex medical and psychosocial services for people with mental disorders which is a basic requirement for the provision of assertive community treatment. As a result of these legislative changes, some of the 146 German health insurance companies started to implement integrated mental health care services along the principles of ACT in collaboration with local mental health service providers. To date, there are only few systematic trials on the effectiveness and cost-effectiveness of these integrated services. Only regional pilot projects have been evaluated and most of these studies are limited by methodological shortcomings.

Within the framework of an analysis of a programme for integrated care (IC) of patients with schizophrenia developed on the basis of the ACT approach Karow et al. concluded that patients who were treated in the ACT programme showed a greater improvement in psychosocial and clinical outcome parameters after 12 months than patients in routine care. This study also showed a reduction in expenses for inpatient treatment with a simultaneous increase in expenses for outpatient treatment and no significant differences in total costs between both programmes were revealed. However, as part of a cost-effectiveness analysis, the authors demonstrated that treatment efficiency (relationship between treatment costs and gain in quality of life) in the ACT group was better than in routine care [9,10].

As result of a mirror design study Fischer et al. concluded that IC reduces inpatient hospitalisation by offering a complex range of services in the outpatient setting. However, there is only a shift from inpatient treatment to outpatient care and total expenditures did not change significantly [11].

Hence, studies conducted in Germany confirm the results of international studies: intensified outpatient services do not contribute to a reduction of psychiatric treatment costs but to increased efficiency of psychiatric treatment. The results of the research Karow et al. [9] suggest that, in these cases, similar effects were achieved irrespective of programme design. However, since only pilot projects have been investigated so far, a verification of these effects and the identification of influencing factors under real life conditions of care are pending.

Also, research needs to address the issue of whether integrated care programmes affect relatives or informal carers living with patients with severe mental disorders. It is assumed that family members and informal carers perceive greater personal involvement in integrated care programmes as positive. On the other hand, there is a possibility that the shift of treatment from the inpatient to the outpatient sector is associated with additional burden for relatives and informal carers.

While most of the earlier contracts on integrated psychiatric care were limited to particular regions, one of the larger health insurance companies started in 2009 to enter into identical contracts named “Network for mental health” (NWpG) with different service providers in different regions across the country. Meanwhile other health insurance companies have also initiative NWpG contract projects, and the number of enrolled patients has increased to about 9000.

For this purpose, the focus of this study is the analysis of effectiveness and cost-effectiveness of contracts for integrated care programmes according to NWpG compared to care as usual in real-world surroundings (routine-care) in five regions in Germany. The main outcome criteria are improvement of empowerment as well as improvement of patients’ subjective quality of life. Moreover, the effects of NWpG contracts for integrated care programmes on burden and quality of life of relatives will be evaluated. The results of this study will contribute to a better understanding of the need for outpatient and inpatient psychiatric care and will contribute to the development of current care concepts in Germany.

## Methods/design

### Study design

Although the randomised clinical trial is considered the gold standard for examining the efficacy of mental health care interventions, legal and organisational aspects prevent this design being applied in the current study: The implementation of integrated care programmes took place on the legal basis of the German social code which authorises the health insurances to define the criteria for the eligibility of patients to enrol in integrated care programmes. In addition, the social code gives patients the right to be informed by sickness funds about the opportunity to enrol in an integrated care programme. If a patient fulfils the eligibility criteria, he or she will be informed by the health insurance and asked for the permission to transmit his or her contact data to the local integrated care provider. After the patient gives his or her permission to the sickness fund the local service provider contacts the patient and invites him or her to attend an informative meeting. On the basis of this interview the service provider decides if the patient will be offered enrolment or not. Finally, the patient decides whether to enrol or not.

Therefore, we decided to design this study as a prospective observational controlled trial comparing patients with severe mental disorders enrolled in an NWpG integrated care programme with patients who received standard care in five federal states (Schleswig-Holstein, Northrhine-Westfalia, Berlin, Saxony and Bavaria) covering a broad range of catchment areas with different levels of urbanization, with different local mental health service systems and with different providers of ACT services. Trial duration is 18 months. Data will be collected at baseline and there will be three follow-ups after 6, 12 and 18 months.

Due to preference-based allocation blinding of patients is not feasible. Furthermore, it is not possible to blind the research associates because they would be unblinded at the latest documenting integrated care service use in the Client Socio-Demographic and Service Receipt Inventory (CSSRI).

### Intervention

Participants in the intervention group receive access to integrated care services in addition to routine care. The agreements under the NWpG model for IC comprise the coordination and provision of outpatient psychiatric treatment and care services through mobile multi-professional teams under professional medical supervision.

Remuneration of IC contracts is based on staggered, case-related lump sums: On the one hand, it allows for extensive design flexibility in the programme by the service provider, on the other hand, it also transfers the risk of budget responsibility to the service provider. This risk results primarily from the fact that all costs for treatment in psychiatric inpatient and outpatient clinics must be financed from the lump sum. This so-called ‘malus’ regime does not apply to services by panel psychiatrists or psychotherapists [12].

All services will be documented in the Client Socio-Demographic and Service Receipt Inventory (CSSRI), and therefore, region-specific design options can be incorporated in the final analysis.

Integrated care according to NWpG contracts under § 140a et seq of Volume V of the Social Insurance Code shares the following basic components in each region:

• individual comprehensive case management on the basis of main control centres;

• outreach mental health services including social environment through home treatment;

• low-threshold and need adapted outreach therapeutic care by multi-professional teams under professional medical supervision, including psychiatric nursing, socio-therapy, psychotherapy, psycho-education and crisis intervention;

• guarantee for 24-hours crisis support 7 days a week;

• provision of retreat areas for crisis support outside hospital settings;

• continuous cooperation with patients, relatives and legal representatives to activate existing resources and to strengthen self-help potentials (empowerment) of those affected [12,13].

### Care as usual

The range of offers for patients in the control group (CAU) is restricted to standard care. They do not receive access to the services assigned to integrated care.

Main aspects of usual care in Germany include mental health care offered in institutions such as hospitals, day-hospitals, medical practices, medical care units and outpatient clinics of psychiatric institutes. Dependent on disease severity, patients are entitled to receive special therapies such as psychotherapy or occupational therapy.

Patients have access to socio-psychiatric services, contact and counselling centres and self-help groups. In some cases, patients experience additional support from nurses, social workers or crisis support teams. In sum, offers of mental health services are wide-ranging but they vary from place to place.

### Measurement instruments

The primary outcome will be measured by means of the questionnaire for the assessment of empowerment in patients with affective and schizophrenic disorders (EPAS). The EPAS measures empowerment as the patient’s perceived opportunity to control their own living circumstances on five dimensions: “daily living”, “social relationships and sexuality”, “psychiatric treatment”, “hope and self efficacy” and “self esteem”. Additional subscales are available for patients who are employed and for parents of minor children. The EPAS core module has 33 items and a Cronbachs Alpha of .94 was obtained for the total scale [14].

The secondary outcome measures include 1) reduction in psychosocial and clinical impairment using HoNos [Health of the Nation outcome scale] [15-18], 2) improvement of subjective quality of life using WHO-QoL-BREF [World Health Organisation Quality of Life-short version] [19], 3) reduction of unmet needs for psychiatric and psychosocial services using CAN-EU [Camberwell Assessment of Need-European Version] [20,21], 4) increase in satisfaction with psychiatric treatment using ZuF8 [questionnaire to treatment satisfaction] [22,23], 5) reduction of utilisation of stationary and simultaneous increase in utilisation of ambulatory medical and psychosocial services using CSSRI [Client Socio-Demographic and Service Receipt Inventory] [24], 6) reduction of direct and indirect healthcare costs using CSSRI [24], 7) reduction of costs of recovering a “healthy” year of life (QALY) using EQ-5D [Euro Quality of Life – 5 dimensions] [25].

An additional study-specific questionnaire will be used at visit 2, 3 and 4 to gain ancillary information as regards the importance of and the satisfaction with local community mental health services from the patients’ perspective.

There is the following set of outcome measures for relatives and informal carers: 1) Change in perception of burden related to support of patient suffering serious mental illness using IEQ [Involvement Evaluation Questionnaires] [26], 2) change in subjective quality of life using WHO-QoL-BREF [19] and 3) change in satisfaction with psychiatric treatment using ZuF8 [22,23].

### Sample Size calculation

The sample size calculation is performed for the change in the empowerment total score over 18 months. An effect size of f = 0.2 was assumed to be clinically relevant for the within-between interaction of group*time in repeated-measurements ANOVA with two groups and four time-points of measurement.

Based on this effect size, a power of 0.90, and an alpha level of 0.05, a total sample size of n = 350 is needed. Sample size was further estimated based on a drop-out rate of 30 %, according to experience from the ELAN-study [27,28]. Based on this assumption, a total of 500 patients will be recruited for visit 1. The sample size was calculated using G-Power.

All participating patients will be asked to nominate a relative/informal carer who has regular contact with the patient and who supports the patient with regard to disease-related burden. It is expected that relatives/informal carers of about 50 % of patients will participate in the study. Thus, the sample size for persons of reference will be equal to n = 250. Using a repeated-measurements design with four time-points of measurements this sample size will concede the demonstration of an effect with effect-size f = 0.2 and a significance level of alpha = 0.05 with a power greater than 0.75.

### Trial inclusion and exclusion criteria

According to the eligibility criteria for the enrolment in the NWpG care programmes patients will be included in the study if they have been diagnosed as having a mental illness of the categories F20-F29, F30-F39, F40-F48, F50, F60-F62, F68-F69 and F91- F94 after ICD-10 during the last 12 months. Patients with a primary diagnosis of substance use disorder (F10-F19, ICD-10) will be only included if they have a psychotic disorder due to substance use.

In addition, patients should have either had a psychiatric inpatient admission or an ambulatory prescription of antipsychotic, anxiolytic or antidepressant drugs during the last 12 months.

Patients must be in the age range of 18 to 80 years, and patients with a care level are excluded.

Group allocation is performed on the basis of the type of care provision intended for the coming 18 months.

### Trial recruitment process

A total of 500 patients, in detail 50 IC patients and 50 CAU patients in each of the five regions and half as many (250) relatives will be recruited via multiple settings. Recruitment will be organised by local research associates. The patients for the intervention group will be informed about the study mainly at introductory sessions for general registration for integrated care by service providers. Patient recruitment for the control group occurs with the assistance of cooperating psychiatric/psychosocial institutions (e.g. resident physicians and socio-psychiatric services). Moreover, patients who decline integrated care after the introductory session will be asked to participate in the control group. Posters and flyers advertising the study are also distributed at potential recruitment sites (e.g. medical practices).

Cooperating psychiatric health institutions that agreed to inform their patients about the study will exchange personal contact details of patients interested in the study with the associate on a regular basis. Written patient information is about the aim of the study, benefits and risks of participation and the study procedure. Subsequently, the associate contacts the patient and checks patient eligibility. If patients meet all of the inclusion and none of the exclusion criteria, they are selected for the study, and an appointment for visit 1 will be set. At visit 1, patients are asked to nominate a relative/informal carer who can be contacted by study staff regarding participation in the study. Patients should provide detailed information material about the study to the person nominated. If the person is interested, he or she is asked to contact the local research associate. If there is no reply, research associates will call the patient once again.

### Trial consent procedure

Patients interested in the study can choose either to contact the research associates themselves or to sign a written consent form that contact data can be submitted from cooperating psychiatric health institutions to the research associate and that the research associate is allowed to call the patient.

At the beginning of visit 1, patients will be informed once more with verbal and written information regarding the study, and patients are asked to give written informed consent if they agree to participate in this study. This will be done by local research associates.

Patients are informed that they can withdraw at any time without having to disclose reasons.

Registration in the integrated care programme occurs completely independent of patients’ decision to participate in the study or not.

Patients are asked to nominate an informal carer/relative who can be interviewed with patient approval. Participation of relatives/informal carers requires the written consent of the patient and of the person of reference him- or herself.

### Data collection/management process

In the context of this study patients will be interviewed four times, every 6 months: baseline (visit 1), 6 months (visit 2), 12 months (visit 3) and 18 months after visit 1 (visit 4). The first assessment (baseline, visit 1) in the intervention group should be within the first month patients are registered in the integrated care programme. Follow-up windows are defined as plus/minus 4 weeks for the intended time-point (6 months, 12 months or 18 months). There will be a subgroup-analysis eliminating data collected outside these windows.

All clinical interviews will be conducted in rooms of outpatient institutions or at patient’s homes by trained research associates. Research associates are graduates in psychology or nursing. Outcomes are measured by self-report and face-to-face interviews.

There is the same time sequence of assessments for relatives/informal carers as there is for patients. There are also four assessments: at baseline, 6 months, 12 months and 18 months after baseline. Between the patients’ and the relatives’ visits, there should be less than four weeks. Outcomes are rarely measured in face-to-face interviews. Mostly, relatives/informal carers fill out the questionnaires on their own and send them by mail - if necessary specific issues are clarified by phone.

### Quality assurance

All research associates were issued with several standard operating procedures as to good clinical practice, recruitment, realisation of study visits, completion of questionnaires and of study documents, etc. A special training (e.g. training in HoNos evaluation) of the research associates is part of several project meetings. The study coordinator oversees the whole study process by communicating frequently with research associates as regards outstanding issues, checking the data collection procedure and sending queries if necessary. Some on-site monitoring visits are planned. During the whole study process research associates are tested for inter-rater reliability for HoNos using case vignettes.

### Planned analysis

A detailed statistical plan will be developed and put down in writing prior to the analysis of data. Data capturing is performed using IBM SPSS 21. Data analysis will be carried out using IBM SPSS 21, STATA 12 and SAS 9.3.

Baseline characteristics of the study population will be summarised separately within each study group. Categorical baseline variables will be compared between intervention and control group using chi-square tests. The continuous variables will be compared using t-test and Mann–Whitney U test. Descriptive statistics of all outcomes will be provided for all study groups at different time points. Outcomes will be summarised in terms of the total scores, and the proportion of patients improving from baseline.

The main analysis will be conducted on the intention-to-treat (ITT) population.

#### *Measures for bias control*

Adjustment based on the propensity score method will be used to control the selection bias [29]. The estimation of propensity scores will be made on the basis of logistic regression models including independent variables such as medical history, patient’s clinical condition, socio-demographic characteristics and current living conditions [28].

#### *Dealing with missing values*

Missing values will be handled using the method of multiple imputations [30]. Missing data will be imputed 10 times. Then, each of the completed datasets will be analysed using the proposed statistical method. Final results will be drawn from the average analysis of each of the completed datasets using Rubin’s Rule.

#### *Statistical analysis*

The primary outcome is the change in empowerment total score of EPAS over 18 months. Normal distribution is assumed. This will be checked, and if found to be lacking than appropriate solution strategies will be applied. Data analysis for the primary outcome will be performed using analysis of variance with repeated measurements with a time effect, a group effect and an interaction effect between time and study group.

In addition, secondary analysis for the primary outcome will include a per-protocol (PP) approach. The PP population will exclude all subjects in the ITT population who meet any of the following criteria:

– change of study group during study process

– non-compliance or infrequent use of integrated care by volunteers in the IC group

– more than four weeks between registration to integrated care and visit 1 for patients in the IC group

– visit 2, visit 3 and visit 4 were outside initially planned time periods (more than +/− 1 month to intended time points: 6 months, 12 months or 18 months after visit 1)

This additional analysis assesses the maximal intervention efficacy in ideal conditions based on comparable outcome measurements.

For secondary endpoints mixed-effect regression models will be used. The random effect of time as well as the interaction effect of time and study group will be adjusted for propensity scores [28].

Within the frame of health economic evaluation based on the net benefit approach, multivariate regression models for the cost-utility relation as dependent variable will be calculated adjusted for the propensity scores. The cost-utility relation is defined as the ratio of direct and indirect costs and the change in QALYs [31-34]. Change in QALYs will be calculated on the basis of EQ-5D questionnaire. All costs will be calculated from a societal point of view, for that reason total costs of care were considered and not just out-of-pocket payments. Furthermore, production losses due to time off work for those in employment will be included in the analyses.

### Ethical considerations

The informed consent procedure is described in the section “trial consent procedure”.

All patients enjoy freedom of choice as regards their utilisation of health-related services. However, routine care provision is expanded by integrated-care specific services (e.g. hometreatment) for patients registered in IC programmes. No patient group is disadvantaged regarding clinical care. Decisions concerning medical treatment and hospitalisation are performed by independent physicians as required. There are no adverse events expected to be related to the integrated care specific services.

Allocation to study groups depends on decisions of statutory health insurances and on patients’ preferences, completely independent of the study. No costs are imposed on patients, and all patients receive 10 € per visit to cover minor expenses.

The obtained data are strictly confidential.

Source documents are specified only by code. Using that code, data are entered into the electronic database. Source documents are maintained in a locked up cabinet in a room in the bureau of the local research associates or of the research coordinator settled at the University of Ulm. Source documents are scanned and sent by mail to the research coordinator for central data-entry just after the interview. Source documents are collected at study sites over two to three months, and will then be shipped to the research coordinator.

The research coordinator oversees the whole study procedure, and she receives progress reports of the project in each centre, if necessary, to make decisions with regard to recruitment strategies or undesired events.

The study is being conducted in compliance with the Declaration of Helsinki 2013.

It has been approved by the Ethics Committee of the University of Ulm on 3^rd^ May 2013 (application number: 129/13 and by the Ethics Committee of the TU Dresden on 30^th^ September 2013 (application number: EK 259072013).

This trial is registered with DRKS (German Clinical Trials Register) and ICTRP (International Clinical Trials Registry Platform) with the identifier DRKS00005111.

## Discussion

This multi-centre controlled study examines the effectiveness and efficiency of contracts of integrated care in patients suffering serious mental illnesses in routine care surroundings. This is the first independent trial in Germany examining the impact of integrated care programmes pursuant to NWpG contracts on empowerment, quality of life, patient satisfaction and health economic measures.

As a major strength of this study, the effectiveness and the efficiency of integrated mental health care will be observed under real world conditions. Research in real life conditions might be more difficult but it will pay off further effort when it comes to external validity of the results. Since the intervention will be delivered by the local integrated care providers in the regional community mental health setting, it can be expected that service provision varies in spite of the fact that the same basic service components have to be provided in each region. In addition, study regions vary with regard to available standard care services and general living circumstances. Furthermore, the study sample will reflect the full heterogeneity of patients with severe mental illness. This heterogeneity of the real world setting provides the opportunity to examine the impact of a broad range of individual and environmental characteristics on the outcome of mental health care.

To make full use of this potential, high levels of academic rigour will be maintained by the involvement of independent, trained research associates and the application of standardised measurement tools, a strict monitoring and quality control for data collection and management and the application of advanced statistical methods for data analysis.

The preference-based allocation of study participants might be the major target of criticism as it is suspected to introduce a selection bias. Preference-based allocation was mainly chosen due to the legal and organisational framework of NWpG contracts in Germany. But it also holds the benefit of reality proximity of the intervention and thus contributes to external validity of study results. Selection bias will be controlled by means of propensity score adjustment. However, it cannot be guaranteed that all factors possibly causing a selection bias can be measured and included in the analysis.

Standard care is permanently undergoing a change. An example is the inclusion of ever more interventions with a view to reducing hospitalisation. Thus, historical data will not be up to date, and it is imperative to simultaneously examine changes in patients receiving standard care. There will be regional differences in the development of mental health care services, too. However, based on the Client Socio-Demographic and Service Receipt Inventory we will be able to integrate such special regional care features in IC as well as in CAU in the statistical analysis.

## Conclusion

The integrated mental health services examined in this study are currently being tested in various parts of Germany. However, systematic data on effectiveness and efficiency are not available yet. The results of the study will provide information for service providers and purchaser organisations (health care funders) about how integrated care programmes in their present form contribute to the improvement of mental health care.

The focus will be on the ability of IC services to overcome existing deficiencies at the interface between outpatient and inpatient mental health care and their performance from the point of view of patients, health care funders and the national economy. In addition, the study will provide hints to weaknesses of the current NWpG model and options to overcome them.

### Trial status

Currently recruiting status:

N_current_[patients, IC] = 228

N_current_[patients, CAU] = 170

N_current_[persons of reference, IC] = 105

N_current_[persons of reference, CAU] = 58

as of 7^th^ may 2014

## Abbreviations

ACT: Assertive community treatment; CAN-EU: Camberwell Assessment of Need-European Version; CAU: Care as usual; CSSRI: Client Socio-Demographic and Service Receipt Inventory; EPAS: Empowerment of patients with affective and schizophrenic disorders; EQ-5D: Euro Quality of Life – 5 dimensions; HoNos: Health of the Nation outcome scale; IC: Integrated care; IEQ: Involvement evaluation questionnaires; ITT: Intention-to-treat; NWpG: Network Mental Health; PP: Per-protocol; QUALY: Quality-adjusted life years; WHO-QoL-BREF: World Health Organisation Quality of Life; ZuF8: Questionnaire to treatment satisfaction.

## Competing interests

The study was financed by the German Federal Ministry of Health (IIA5 – 2513FSB012). The local service providers of integrated mental health care (NiG Berlin, PTV Sachsen, Brücke SH, Kieler Fenster, Awolysis (Vincentro München), PTV Solingen) agreed to contribute to study financing in terms of non-personnel-costs and travel costs of the local research associates. KH is concurrently employed by PTV Solingen. JW and MJH were employed until the start of the study by NiG Berlin and Awolysis (Vincentro München), respectively. Patient recruitment was conducted in close cooperation with service providers of integrated mental health care and other professionals from the outpatient setting. There are no other relationships or activities that could appear to have influenced the submitted work.

## Authors’ contributions

RK, TB, MS with others developed the study design. All authors are involved in conducting the study. ASS is accountable for data preparation, ASS and RK will be responsible for statistical analysis. ASS drafted the first manuscript, RK supervised her. All others commented and contributed to successive drafts. All authors read and approved the final manuscript.

## Pre-publication history

The pre-publication history for this paper can be accessed here:

http://www.biomedcentral.com/1471-244X/14/163/prepub
